# An Integrated Approach to Nuclear Risk Governance in Decommissioning with a Major Focus on Stakeholder Risk Philosophies

**DOI:** 10.1007/s00267-026-02575-1

**Published:** 2026-08-05

**Authors:** Sally Njogu, Leslie Mabon, Gareth Neighbour

**Affiliations:** School of Engineering and Innovation, The Open University, Milton Keynes, UK

## Abstract

This paper addresses how nuclear risk governance can ensure long-term safety, societal trust and ethical responsibility in managing nuclear waste under conditions of uncertainty. This challenge is shaped by increasing policy demands for transparency, sustainability and meaningful public engagement following major nuclear incidents. Existing approaches to risk governance draw on a range of theoretical perspectives, spanning technical, social and ethical domains. However, these are often applied in isolation, resulting in fragmented governance models that fail to capture the full socio-technical complexity of nuclear risk. To address this gap, the study critically synthesises key perspectives, including technical risk-based approaches, social and cultural theories of risk perception, decision and economic models and ethical frameworks concerned with justice and responsibility. The findings show that while each perspective offers valuable insights, none is sufficient on its own to achieve comprehensive governance, understood as an integrated approach that accounts for technical, social, ethical, institutional and uncertainty-related dimensions of risk. In response, the paper develops a pluralistic approach that brings these perspectives together into a coherent framework. This enables more adaptive, inclusive and context-sensitive risk governance. The study contributes a conceptual stakeholder-oriented framework to support policymakers in designing governance systems that are not only technically robust but also socially legitimate and ethically grounded in the context of nuclear waste management and decommissioning.

## Introduction

As of June 2024, 210 nuclear reactors worldwide were permanently shut down, with the United States recording the most shutdowns, with 41 units. The United Kingdom follows, with 36 reactors, and Japan with 27 reactors (IAEA, 2024). All these reactors are in various stages of decommissioning. Over recent years, nuclear decommissioning has been characterised by a range of persistent challenges, including technical uncertainty, cost escalation, regulatory complexity and stakeholder-related controversies, which have collectively impeded progress across multiple projects (IAEA, 2018; NAO, 2020; OECD-NEA, 2016). Consequently, decommissioning governance has emerged not only as an engineering and regulatory concern, but also as a critical policy issue linked to public legitimacy, long-term sustainability and societal trust in nuclear institutions. As nations continue to expand or retire nuclear infrastructure in response to climate, energy security and decarbonisation agendas, the governance of decommissioning processes has become increasingly important within international nuclear policy and practice.

Nuclear decommissioning is not solely a technical process but also includes an intense socio-political and ethical challenge. Communities often perceive higher risks than regulators acknowledge, driving opposition and demands for stricter controls (Slovic, 2000). Debates persist over intergenerational fairness in waste management, with questions remaining about whether permanent disposal or retrievable options best serve future generations (Shrader-Frechette, 1991; Tondel and Lindahl, 2019; Yasuda et al., 2024). These debates reflect broader tensions within risk governance scholarship concerning the relationship between expert knowledge, public values, uncertainty and legitimacy in decision-making processes.

Accidents such as Fukushima and Chornobyl amplify distrust in both administrative bodies and scientific institutions, making siting decisions and decommissioning work highly contested despite low estimated risks. Disagreements arise from cost allocation among operators, governments and taxpayers, which is further complicated by long timelines and economic instability, including financial fluctuation and fiscal variation, such as inflation (World Nuclear Association, 2022). Scientific uncertainties hinder progress, ranging from geological stability to the impacts of climate change, e.g. nuclear waste disputes over geological disposal versus retrievable storage (OECD-NEA, 2019). Moreover, the introduction of robotics and artificial intelligence (AI) raises concerns about reliability, safety and cost-effectiveness (OECD-NEA, 2019). Each nuclear licensed site’s unique legacy conditions further complicate consensus on defining a ‘safe’ end-state among regulators, operators and local stakeholders. Radiological risk, encompassing impacts on human health, safety and the environment, constitutes the primary distinguishing factor across all stages of the nuclear lifecycle, with financial, investment and project management risks arising largely as secondary consequences of managing and mitigating these hazards (IAEA, 2018).

The approach to governance in nuclear decommissioning varies from one nation to the next for these reasons. There are common aspects, such as the determination of objective risk, but the thresholds and classifications may vary. Nuclear risk governance, for the purposes of this paper, refers to the ensemble of institutions, policies and processes through which nuclear risks are assessed, managed and communicated across technical, social and political domains (adapted from Renn, 2008). This definition situates the present study within broader theoretical debates concerning how risk is conceptualised, legitimised and operationalised across competing stakeholder groups and institutional contexts.

Objective risk, as used in the nuclear industry, is the factual, measurable risk, often defined as the probability of an event or scenario occurring and its potential consequences. It is the potential for an event or action to lead to negative consequences, calculated as a combination of the likelihood (or probability) of an adverse event and the severity (or impact) of its consequences (Möller, 2012). In addition, the approach will involve a range of principles, paradigms and other philosophies. From a soft systems thinking perspective (Checkland, 1989), multiple perspectives co-exist in an organised system regarding the risk and this complexity is rarely explicitly considered, thereby the role of informal and subjective influence that holds the power and could leverage force on the directionality of the whole organisational system, yet it is fundamental to societal acceptance of the risk. Here, the demonstration seeks to provide a more integrated approach to risk assessment. Exploring questions about the nature of risks, the social construction of risk issues and the cultural differences in conceptualising and understanding risk has influenced contemporary philosophical thinking and social science research (Renn, 2008).

Conflicting risk philosophies, including the epistemologies (knowledges), axiologies (values and ethics) and teleologies (motivations and interests) of different stakeholders, create misalignment in how individuals and stakeholder groups identify, assess and respond to these risks. This misalignment can lead to confusion, inefficiency and missed opportunities. For instance, different risk tolerances (e.g. risk-averse vs. risk-seeking) can lead to inconsistent approaches when making critical decisions. Accordingly, one stakeholder group may prioritise aggressive growth despite high risks, while another may focus on minimising exposure, leading to conflicting strategies. Differing views on acceptable risk levels can also lead to misunderstandings or tension among stakeholders. These, in turn, cause miscommunications that can slow down decision-making or cause essential risks to be overlooked (IAEA, 2021). When different stakeholders follow conflicting risk frameworks, it can lead to redundant processes or gaps in risk management (OECD-NEA, 2019). If there is no unified risk philosophy, it becomes harder to assign responsibility when risks materialise. Conflicting philosophies can create a culture of uncertainty, leaving different stakeholder groups unsure how to approach risks. It may foster an environment where innovation is either stifled (due to excessive caution) or reckless (due to inadequate safeguards) (Renn, 2008; Sandin, 1999). These tensions underpin a growing debate in the governance literature over whether existing stakeholder engagement models are sufficiently capable of addressing deep value pluralism and epistemic diversity in high-risk technological systems, such as nuclear decommissioning.

This paper focuses on synthesising stakeholder philosophies in the literature on nuclear site decommissioning to help conceptualise a more balanced, inclusive and adaptive approach to risk governance that better addresses uncertainty, builds trust and improves overall outcomes. Therefore, our demonstration proposes a novel governance framework, termed the Pluralistic Approach to risk governance, designed to integrate diverse risk philosophies and guiding principles into the practice of nuclear risk governance. In doing so, this study contributes to existing debates by moving beyond procedural stakeholder inclusion towards a substantively pluralistic framework that explicitly incorporates diverse forms of knowledge, values and motivations into governance structures. Defining the proposed framework necessitates a systematic categorisation of nuclear stakeholders and an articulation of the dominant risk philosophies underpinning each group’s perspective, as presented herein. For this paper, we define classification as involving a formalised, predefined system of organisation, while categorisation is any grouping based on similarity.

While institutional frameworks such as those proposed by the International Atomic Energy Agency and International Risk Governance Council (IRGC) (IAEA, 2021; Renn, 2008) advocate stakeholder inclusion, they primarily reflect procedural forms of pluralism. In contrast, this study advances a substantive pluralistic approach that recognises and explicitly integrates diverse epistemologies (knowledges), axiologies (values and ethics) and teleologies (motivations and interests). The approach of this study treats expert and stakeholder perspectives as contextually rational rather than viewing experts as ‘rational’ and other stakeholders as ‘irrational’. This study, therefore, treats these different perspectives as epistemically complementary rather than hierarchical. This is then integrated into a coherent framework using five principles. Procedural approaches are concerned with the process, rules and methods used in decision-making, while substantive approaches focus on the quality, content and ethical validity of outcomes, rather than the process alone.

## The Illustrative Case about Nuclear Stakeholders

The Sellafield nuclear site, for example, has faced substantial cost overruns, with decommissioning expenses escalating to £136 billion (2025) from a previous figure of £115 billion (2023-24 prices), representing an increase of £21 billion, or approximately 18.8%, according to a report by the UK Parliament’s Public Accounts Committee (PAC) in June 2025. Unrealistic budgeting and differing risk assessments have contributed to project delays and financial inefficiencies (Lawson and Isaacs, 2025). The decommissioning process at the San Onofre Nuclear Generating Station, in the USA, meanwhile, was hindered by regulatory interventions due to safety concerns, resulting in delays and legal disputes (Lochbaum, 2014).

This situation highlights how differing risk perspectives between operators and regulators can hinder progress (Weart, 1988). Following the Fukushima disaster, Japan’s shift back towards nuclear energy has sparked public opposition and accusations of political hypocrisy. Conflicting risk philosophies between governments and the public have led to disputes and a decline in trust (ONR, 2014). Möller (2012) and Rosa (1998) noted that although the traditional model of scientific investigation is essential for risk analysis, particularly in identifying and estimating risks, it falls short in addressing risk evaluation, mitigation and communication. Due to this, Rosa (1998), Renn (1995, 2008) and Stern and Fineberg (1996) were all in consensus on the importance of continuing to define risk as an inherently interdisciplinary activity and due to its interdisciplinary position, they were also in consensus on how important it would be to devise procedures for better governance of these risks.

Stakeholders tend to exhibit unique needs and expectations, varying across economic impact, environmental efficiency, technologies, political aspects and more (Banerjee and Bonnefous, 2011). The term ‘stakeholder’ refers to individuals, groups, or organisations with a personal stake, involvement, or investment in the relevant activities, also known as interested or concerned parties (Ahmed et al., 2020). The IAEA Nuclear Energy Series No. NG-T-1.4, provides a comprehensive guide to increase the effectiveness of stakeholder involvement across various stages of the life cycle of nuclear facilities, including construction, operation, radioactive waste management, decommissioning and more (IAEA, 2021). This work emphasises the utilisation of updated methods to implement stakeholder involvement programmes. Additionally, a report from the International Nuclear Safety Group (INSAG-20) offers guidelines for stakeholder involvement on issues related to nuclear activities (IAEA, 2021; Ahmed et al., 2020). Understanding stakeholder philosophies is a prerequisite for integrating their perspectives into the governance process, enabling more inclusive, transparent and resilient decision-making. The varied reasons for including stakeholders in the governance process, as recorded by Fiorino (1990) and Wesselink et al. (2011), are summarised below.

- **Normative Reasons**: Empower stakeholders to express their concerns, values and priorities. Supports inclusivity and democratic decision-making.
- **Pragmatic Reasons**: Enhance legitimacy and gain stakeholder buy-in for processes or decisions. Builds trust and acceptance among stakeholders.
- **Substantive Reasons**: Leverage the value of lay knowledge alongside expert knowledge to improve outcomes. Improves the quality, relevance and soundness of research.

Effectively involving stakeholders in nuclear power project-related activities requires meticulous identification of the primary parties involved, as these actors hold diverse standpoints and may have competing interests that influence their interpretation of risk and participation in governance processes (Renn, 2008). Some stakeholders may exhibit limited direct interest, yet their influence can be significant. This is often due to underlying power dynamics and the uneven distribution of resources, such as political authority, regulatory control, financial capital, technical expertise, or media access, that enable certain actors to shape decision-making processes and outcomes even without strong direct engagement (Lukes, 2005). Consequently, influence in governance contexts is not solely determined by expressed interest, but also by structural positions within broader institutional and socio-political networks, where stakeholder salience is shaped by the interaction of power, legitimacy and urgency (Mitchell et al., 1997; Renn, 2008). It is therefore imperative to recognise these influential stakeholders, even when their interests appear modest, to ensure comprehensive engagement and to address pivotal concerns that may otherwise remain implicit but highly consequential. This approach acknowledges that impact and influence are not always directly correlated with the level of interest. De Vente et al. (2016) and van Vleit et al. (2020) noted that strategic engagement with all relevant parties is essential for the success and acceptance of nuclear power projects, underscoring the importance of a holistic and inclusive stakeholder involvement strategy. According to Ahmed et al. (2020) and Schweitzer and Mix (2021), nuclear stakeholders can be categorised in various ways, with illustrative examples provided in Tables 1 and 2 below. It is important to emphasise that these categorisations are not exhaustive or exclusive; rather, they reflect some of the most widely adopted and established approaches within nuclear governance, while recognising that alternative frameworks may be applied depending on contextual and analytical requirements.

**Table 1:** Categorisation of Nuclear Stakeholders (adapted from Ahmed et al., 2020)

Stakeholder Group	Description	Examples
**Project Developers (P)**	Individuals and organisations responsible for the ownership, investment, operation and management of nuclear sites and facilities.	Owners of nuclear sites and facilities, investors, operational staff and management.
**Regulators (R)**	Entities that ensure compliance with nuclear regulations and oversee industry practices.	Supervisory authorities, industry bodies and local & National Governments.
**Co-operators (C)**	Organisations and groups that work alongside or provide support to nuclear projects.	Trade unions, waste management organisations, local contractors, nuclear and non-nuclear industrial companies, local entrepreneurs, NGOs and non-regulatory ministries.
**Indirect Influencers (I)**	Groups not directly involved in nuclear projects but significantly impacting public perception and acceptance of nuclear activities.	General public, neighbouring countries, indigenous peoples, landowners, scientists, educators and students, universities, tourists, archaeologists, historians, museums, archives, media, health workers, lobby groups, religious and non-religious organisations, political leaders, experts’ communities, intellectual bodies, social media celebrities, millennials.

**Table 2:** Categorisation of Nuclear Stakeholders (adapted from Schweitzer and Mix, 2021)

Stakeholder	Description	Examples
**Oppositional Stakeholders (O)**	Stakeholders are actively opposed to nuclear energy and activities.	Anti-nuclear activists.
**Monitoring Stakeholders (M)**	Stakeholders are responsible for providing information, oversight and expertise to the public.	Organisations experts and media.
**Normative Stakeholders (N)**	Individuals directly involved in the nuclear industry as employees or representatives.	Workers, industry representatives.

## Theoretical Background on Risk Philosophies

Beck (2009) proposes the concept of the Risk Society, highlighting how modernisation produces new risks that undermine its own foundations, compelling societies to confront the unintended consequences of technological progress. This process, termed reflexive modernisation, refers to the ‘boomerang effect’, in which unforeseen outcomes of industrial and scientific advancements backfire on society, forcing adaptation (Wimmer and Quandt, 2006). In this context, governance must continuously reflect on and manage emerging risks, making adaptability central to policy development. Applied to nuclear energy, Beck’s framework underscores the transboundary nature of nuclear risks and advocates international cooperation and governance (IAEA, 2021). It shifts focus beyond technical solutions to include social, cultural and ethical dimensions, promoting continuous learning and stakeholder engagement as essential for addressing complex nuclear challenges (OCED-NEA, 2019).

However, critics argue that this risk-centric approach may overshadow nuclear energy’s benefits, such as its role in decarbonisation (De Groot et al., 2013; Guo et al., 2024). Reflexive governance also faces practical challenges: balancing safety with energy demands, managing diverse stakeholder interests and avoiding regulatory uncertainty that could discourage investment (Rowe and Frewer, 2000; De Vente et al., 2016). Public engagement remains contentious, as illustrated by Germany’s post-Fukushima nuclear phase-out, driven by public opposition and ethical considerations (Kochskämper et al., 2016; Low, 2020; Polleri, 2021). Ultimately, Beck’s theory calls for dynamic, transparent and flexible nuclear governance, enabling societies to anticipate and respond to emerging risks while addressing ethical responsibilities for past and present hazards (Giddens, 1999). Yet, excessive reflexivity risks slowing decision-making and complicating long-term nuclear strategies.

The section below synthesises literature on different stakeholder risk philosophies, including the stakeholder epistemologies, axiologies and teleologies and how they significantly shape the nuclear sector, influencing policy decisions, public acceptance, investment and technological development. Governments and regulators, for example, often adopt a precautionary approach, prioritising public safety and environmental protection over economic or operational efficiency. For example, after the Fukushima disaster in 2011, several countries, most notably Germany, implemented stricter regulations or sought to phase out nuclear power entirely. Germany’s *Energiewende* policy led to a rapid transition away from nuclear energy in favour of renewable alternatives due to public safety concerns, thereby initiating the process of decommissioning most of its nuclear sites.

### Stakeholders’ Perspectives

#### The Realist Perspective

The Realist Perspective is a foundational framework for understanding risk. According to Rosa (1998), it comprises two main categories: Ontological Realism (OR) and Epistemological Hierarchism (EH). Both assume that risks exist objectively and can be quantified independently of human perception, typically through probabilistic and statistical methods.

OR asserts that an objective world exists and can be known, albeit imperfectly. EH, while acknowledging the fallibility of all knowledge, maintains that some forms, particularly scientific and technical, are more reliable than others. This perspective underpins much of nuclear risk assessment, emphasising objective measurement through models that quantify radiation levels, decay rates and failure probabilities (Renn, 2008). OR reinforces the notion that nuclear risks are real and measurable regardless of public opinion, while EH legitimises prioritising scientific expertise in decision-making. Consequently, risk governance strategies informed by realism tend to be highly structured and evidence-based, resulting in rigorous safety protocols, reactor design standards and emergency response plans (Perrow, 1984).

However, Stirling (2007), Taebi and Roeser (2015) and Wynne (1992) note that the above technical approach may overlook the social and psychological dimensions of risk perception, which struggle with the uncertainties inherent in nuclear systems. By focusing predominantly on measurable risks, realism can overlook cultural factors that influence public trust and acceptance, potentially leading to opposition or governance challenges.

#### Social Constructionist Perspective

The Social Constructionist approach views risk as a product of social, cultural and individual factors rather than a factual or verifiable reality. Rosa (1998) argues that this perspective emphasises perception in defining what is considered risky. For strong constructivists, there is little distinction between perceived reality and reality itself (Stallings, 1995).

According to Wynne (1992), risks are embedded within social relations and shaped through the continual negotiation of social identities. This approach recognises that societal perceptions, cultural norms and historical contexts significantly influence how nuclear dangers are understood. Consequently, it advocates for inclusive and transparent governance that takes into account diverse stakeholder experiences and values (Hogg and Brown, 2019; Hughes, 2012). Engaging stakeholders and the public in dialogue, rather than relying solely on expert assessments, can build legitimacy and foster trust (Wynne, 1992). Such engagement is crucial in democratic contexts, where nuclear policy requires broad social acceptance.

However, as Rosa (1998) and Flynn et al. (2001) note, policies based on socially constructed risk can be challenging to implement due to conflicting perceptions among stakeholders. Jasanoff (1998) further observes that while this approach enhances inclusivity, it complicates governance by introducing diverse and sometimes incompatible risk assessments. As a result, decision-making often requires balancing scientific evaluations with public concerns to achieve socially acceptable and technically sound outcomes.

### Theories on Stakeholders

#### Cultural Theory of Risk

Douglas and Wildavsky (1983) proposed a Cultural Theory of Risk where risk is socially constructed and defined by societal values, with perceptions shaped by cultural worldviews. This theory provides a comprehensive framework for understanding risk, encompassing elements such as objectivity, science, rationality and public perception (Golding et al., 1992). It identifies four cultural biases that influence how groups interpret and respond to risks: Individualist, Egalitarian, Hierarchical and Fatalist.

Cultural Theory has been widely applied to explain variations in responses to environmental and technological risks across different societies (e.g. Furedi, 2007). By considering these cultural orientations, policymakers can design more inclusive and effective risk governance strategies (Tansey and O’Riordan, 1999). For instance, egalitarian groups often oppose nuclear power due to concerns about environmental justice, while hierarchical groups tend to trust expert regulation and institutional control (Rayner, 1992). Understanding these cultural biases allows risk communication and engagement strategies to be tailored to specific worldviews, improving legitimacy and acceptance.

However, cultural perspectives are often deeply rooted, making consensus-building between groups with opposing values particularly challenging (Hughes, 2012).

#### Psychometric Paradigm

The Psychometric Paradigm examines the psychological dimensions of risk, focusing on how people perceive and emotionally respond to hazards (Slovic, 2000). It highlights cognitive biases, such as the availability heuristic, where people overestimate risks that are easily recalled and the affect heuristic, which influences judgments by emotions. Key components include dread risks, which evoke fear due to catastrophic potential, fatal consequences and lack of control and unknown risks, which are unfamiliar, poorly understood and often have delayed or invisible effects (McGuire et al., 2010; Sung et al., 2022). In the context of nuclear risk, these factors contribute to strong public reactions to issues like radiation exposure, nuclear waste and accidents (Slovic, 1987).

The paradigm explains why nuclear risks often trigger anxiety and distrust, emphasising that emotional responses can significantly shape public attitudes. Recognising these psychological drivers enables policymakers to design targeted communication strategies, provide transparent information and counter misinformation (Hooker et al., 2017). While this approach supports public education and engagement, it also calls for balancing risk communication with respect for legitimate concerns, rather than dismissing them as irrational fears (Fischhoff et al., 1978).

#### Complex Systems Theory

Complex Systems Theory views risk as arising from interactions within interconnected and interdependent systems, emphasising a holistic approach to understanding and managing uncertainty (Funtowicz and Ravetz, 1994). Unlike ordinary complex systems, which maintain dynamic stability until overwhelmed, emergent complex systems, such as human societies or nuclear infrastructures, exhibit non-linearity, interdependencies and adaptive behaviour, making reductionist approaches ineffective (Norberg and Cumming, 2008).

Nuclear risks exemplify emergent complexity, as failures can cascade across technical, environmental and social dimensions (Ramana, 2011). This perspective prioritises resilience and robustness in governance frameworks, promoting strategies like defence-in-depth, adaptive safety mechanisms and comprehensive risk assessments that account for systemic vulnerabilities (Perrow, 1984; Dekker, 2011).

However, predicting outcomes in such systems remains challenging due to high uncertainty, non-linear interactions and emergent properties, requiring continuous monitoring and adaptive policies (Funtowicz and Ravetz, 1994). While Complex Systems Theory offers valuable insights for designing flexible and resilient governance, its practical implementation is limited by the technical expertise and sophisticated modelling required to address interconnected risks (Ramana, 2011).

#### Economic and Decision Theory

Economic and Decision Theory, as described by Freudenburg and Pastor (1992), is grounded in rational choice, assuming that individuals and organisations make decisions to maximise expected utility. It is commonly operationalised through cost-benefit analysis (CBA), which evaluates trade-offs between risk mitigation costs and the benefits of reduced risk (Butt et al., 2008). In the nuclear sector, CBA informs decisions such as the economic feasibility of constructing new reactors, decommissioning strategies and safety enhancements post-Fukushima (ONR, 2014; IAEA, 2011; Figueroa, 2013).

The approach provides a quantitative framework for decision-making, offering measurable outcomes and integrating uncertainty through probabilistic risk assessments (Arvai and Froschauer, 2010; Riesch, 2012). Trade-off analysis helps stakeholders balance competing priorities, such as cost versus safety or short-term expenditure versus long-term benefits, while its transparency supports accountability by documenting decision rationales (Sjöberg and Drottz-Sjöberg, 2009).

However, significant limitations exist. Many costs and benefits, such as externalities like environmental impacts, public health and intergenerational equity, are difficult to monetise (Agard et al., 2014). Predicting the costs of low-probability, high-impact events, such as major accidents, is inherently uncertain. Furthermore, prioritising economic efficiency can overlook ethical responsibilities, like safeguarding public safety (Hooker et al., 2017). Finally, decision theory often oversimplifies complex interdependencies and social dynamics, reducing its suitability for highly uncertain, emergent nuclear systems.

### Disposition Regarding Stakeholders

#### Precautionary Principle

The Precautionary Principle advocates preventive measures in the face of uncertainty, particularly when risks may cause severe or irreversible harm. It asserts that scientific uncertainty should not delay protective action, a principle widely applied in environmental and public health policies (Renn, 2008; United Nations, 1992).

In nuclear governance, historical accidents underscore its relevance. The Chornobyl disaster in 1986, driven by inadequate reactor design and poor safety culture, demonstrated the cost of failing to implement precautionary measures (ICRP, 2020; Wetherholt and Heimann, 2010). Similarly, the Fukushima Daiichi accident (2011) revealed insufficient preparation for extreme natural hazards, prompting global reforms such as enhanced flood protection and improved emergency response systems, especially in the context of beyond design basis events (IAEA, 2011). The principle also shapes long-term strategies, as seen in deep geological waste repositories like Onkalo (Finland) and Forsmark (Sweden), designed to prevent contamination for millennia (IAEA, 2018). By promoting robust safety protocols and stricter regulations, the precautionary principle strengthens nuclear risk governance.

However, overly conservative application can impede innovation and delay the development of beneficial technologies (Sandin, 1999). Balancing precaution with scientific advancement remains a critical governance challenge, especially in regions reliant on nuclear power for energy security and economic stability.

#### Ethical and Moral Frameworks

Ethical and moral frameworks emphasise justice, equity and the protection of vulnerable populations in risk governance (Renn, 2008; Möller, 2012). They stress the ethical duty to minimise harm and ensure a fair distribution of risks and benefits, particularly in areas such as nuclear waste disposal, energy policy and disaster response (Graham et al., 2011; Alzahrani et al., 2023; Ohtomo et al., 2014). These frameworks extend to decisions on nuclear power generation, weapons development and long-term waste management.

Historical cases illustrate the role of ethics in nuclear governance. The Yucca Mountain Repository project in the U.S. faced opposition from Indigenous groups and local residents on grounds of environmental justice and intergenerational equity, ultimately stalling due to ethical and political resistance (Weart, 1988; Graham et al., 2009). Similarly, the transition to low-carbon energy raises ethical dilemmas: proponents argue that nuclear energy is a moral imperative for reducing emissions, while critics highlight concerns over safety, waste and justice (OCED-NEA, 2019). France’s reliance on nuclear power reflects this ethical tension, prioritising climate mitigation while grappling with unresolved waste issues (Banerjee and Bonnefous, 2011). These frameworks challenge who bears nuclear risks and who benefits, advocating for fairness in siting, waste storage and disaster preparedness (Shrader-Frechette, 1991). They require policymakers to consider marginalised communities, intergenerational responsibilities and global equity in nuclear governance (O’Neill, 1993; Taebi and Roeser, 2015). However, balancing ethical imperatives with technical feasibility and economic constraints remains difficult. Tensions arise when precautionary principles or cost-benefit analyses conflict with moral obligations, underscoring the need to prioritise values transparently in decision-making.

### Methodology and Logical Model

This paper builds upon a conceptual synthesis methodology (Jaakkola, 2020), drawing upon principles of integrative literature review and theory-building research to develop a coherent analytical framework for stakeholder engagement in nuclear governance. Rather than testing hypotheses empirically, the objective is to generate conceptual understanding by synthesising diverse theoretical perspectives into a unified interpretive framework. Such an approach is appropriate where existing theories provide complementary but fragmented explanations of complex socio-technical phenomena and where conceptual integration can advance both theoretical clarity and practical application (Torraco, 2005; Snyder, 2019; Jaakkola, 2020).

The analysis is informed by an interpretive and comparative examination of existing stakeholder, governance and risk literature. Each perspective is critically evaluated with respect to its underlying epistemological assumptions, treatment of uncertainty and implications for stakeholder engagement in high-risk technological settings such as nuclear decommissioning. Rather than treating these perspectives as competing explanatory models, the analysis considers them as reflecting different normative orientations towards risk and decision-making. This interpretive stance enables a comparative assessment of their respective strengths, limitations and complementarities while recognising that complex governance challenges cannot be adequately understood through a single theoretical lens.

Consistent with established approaches to qualitative conceptual synthesis, recurring themes across the literature are identified through iterative comparison, abstraction and thematic integration (Thomas and Harden, 2008; Snyder, 2019). The purpose of this process is not statistical generalisation but theoretical abstraction, allowing higher-order conceptual patterns to emerge from multiple disciplinary perspectives. The resulting stakeholder risk philosophy framework, therefore, represents an analytical construct that categorises stakeholder orientations into six idealised archetypes. These archetypes should be understood as heuristic devices rather than empirical classifications, providing an interpretive lens through which stakeholder positions and governance dynamics may be analysed.

Following the development of the stakeholder risk philosophy framework, a second stage of conceptual integration combines this framework with the two established stakeholder categorisation approaches presented in Tables 1 and 2. Integrative theory-building research emphasises the value of reconciling complementary conceptual models in order to improve explanatory power while preserving the strengths of existing theoretical traditions (Torraco, 2005; Jaakkola, 2020). Accordingly, the three frameworks are comparatively examined to identify areas of structural, functional and philosophical convergence and divergence (Tables 1, 2 and 4). This comparative synthesis provides the basis for a consolidated stakeholder categorisation that maintains the empirical and institutional grounding of existing approaches while extending them by explicitly considering normative and epistemological dimensions.

The final stage of the methodology employs normative conceptual analysis to derive the foundational principles of a pluralistic governance approach. Building on the integrated stakeholder framework, relevant literature on governance theory, risk perception, ethics and pluralism is comparatively interpreted to identify higher-order principles that transcend individual stakeholder orientations. The analytical focus is on recurring themes of uncertainty, legitimacy, participation, value diversity and knowledge integration, thereby allowing common governance principles to be abstracted from differing philosophical positions. This process reflects established approaches to conceptual theory development, in which explanatory constructs are progressively refined into broader normative frameworks that inform both scholarly analysis and practical governance (Jaakkola, 2020).

Overall, the methodological approach reflects a progression from conceptual synthesis to theoretical integration and the development of a normative framework. In this case, conceptual research contributes through analytical coherence, theoretical integration and explanatory insight rather than through empirical measurement (Gilson and Goldberg, 2015). The resulting framework is therefore intended as a theoretically grounded analytical tool for interpreting stakeholder diversity and supporting more pluralistic governance and engagement strategies within nuclear decommissioning contexts.

## Logic Result: Strengths and Weaknesses of Stakeholder Approaches

The various philosophies of risk outlined in the approaches above play a crucial role in shaping nuclear risk governance by influencing how risks are assessed, managed and communicated to the public. Due to the high-stakes, complex nature of nuclear risks, including potential catastrophic consequences, long-term environmental impacts and profound public fear, philosophies offer distinct frameworks and strategies that can guide nuclear risk management. While traditional stakeholder mapping typically focuses on power, interest, or influence (Freeman, 1984; Reed et al., 2009), risk perception literature demonstrates that decision-making and conflict often stem from philosophical differences in risk tolerance and mitigation strategies (Renn, 2008; Slovic, 2010).

The philosophies discussed below represent distinct yet interrelated approaches to risk, shaped by differences in institutional roles, decision-making responsibilities and underlying psychological and ethical drivers. The stakeholder-based risk philosophies presented in Table 3 below can be understood as applied expressions of the theoretical perspectives outlined earlier in Section 3. Table 3 extends the existing risk governance literature by explicitly articulating the derivation logic through which theoretical perspectives translate into stakeholder-specific risk philosophies. The risk philosophies represented here reflect only the dominant or overarching tendencies associated with stakeholder groups and should not be interpreted as fixed or universally held positions. Risk attitudes are context-dependent, fluid and internally diverse, with individuals and communities often drawing upon multiple, overlapping and sometimes contradictory perspectives across different issues and decision-making contexts (McGuire et al., 2010; Renn, 2008). While the categorisation identifies dominant orientations associated with particular stakeholder groups, it is recognised that, in practice, these actors draw upon multiple and overlapping theoretical logics. For example, regulators may incorporate not only realist and precautionary principles but also considerations of public perception and political accountability. Similarly, industry actors may engage in precautionary and safety-oriented practices alongside economic optimisation, while non-governmental organisations may utilise scientific evidence alongside socially constructed interpretations of risk. As such, the classifications proposed here are best understood as indicative tendencies shaped by institutional roles and contextual constraints, rather than fixed or mutually exclusive categories. Furthermore, the ‘derived logic’ linking theoretical perspectives to stakeholder philosophies is presented in non-deterministic terms; the relationships described within the framework should be interpreted as tendencies influenced by additional factors, such as trust, institutional credibility and historical context.

**Table 3:** Translation of Theoretical Approaches to Risk to Philosophies of Risk Through Derived Logic

Risk Philosophy	Stakeholder Bodies	Theoretical Foundations	Logic for translating Theoretical foundations to institutional philosophies	Resulting Philosophy
**Realist/Precautionary**	Regulators & Government	Realist Perspective (OR + EH); Precautionary Principle; Elements of Risk Society	Ontological realism holds that nuclear risks are objectively real and measurable. Epistemological hierarchism legitimises scientific expertise and regulatory authority. The precautionary principle introduces conservatism in the face of uncertainty. Risk society adds awareness of systemic and long-term consequences.	Risk is real, measurable, but uncertain; therefore, it must be managed cautiously using evidence-based regulation.
**Risk-Balancing**	Industry & Operators	Economic and Decision Theory; Realist Perspective; Elements of Complex Systems Theory	Rational choice theory promotes cost-benefit optimisation. Realism provides the quantitative tools (such as probabilistic risk assessment). Complex systems thinking acknowledges constraints but still requires operational decision-making.	Risk is inevitable and manageable, so it must be optimised between safety, cost and performance.
**Risk-Averse**	Communities & NGOs	Psychometric Paradigm; Social Constructionist Perspective; Cultural Theory of Risk	Psychometric theory explains fear, dread and perceived lack of control. Social constructivism emphasises trust, identity and lived experience. Cultural theory explains differences via worldviews (e.g. egalitarian scepticism of nuclear power).	Risk is socially and emotionally amplified, leading to a preference for caution, opposition, or alternatives.
**Risk-Intolerant**	Safety Inspectors & Emergency Responders	High Reliability Organisation (HRO) theory; Precautionary Principle; Elements of Realism	Complex systems and HRO thinking highlight the potential for catastrophic cascades. Precaution reinforces minimising even low-probability risks. Realism ensures risks are treated as technically real and critical.	Risk is potentially catastrophic; even small failures are unacceptable (with near-zero tolerance for error).
**Zero-Risk/Principle-Based**	Ethics Committees & Policy Advocates	Ethical and Moral Frameworks; Social Constructionism; Elements of Precautionary Principle	Ethical frameworks prioritise justice, rights and intergenerational equity. Social constructivism allows risk to be framed as morally unacceptable rather than merely technical. Precautions help prevent irreversible harm, regardless of cost.	Risk is normatively unacceptable, leading to principle-driven rejection or strict limitation.
**Global Risk-Mitigation**	International Organisations (e.g. International Atomic Energy Agency)	Risk Society; Complex Systems Theory; Realist Perspective	Risk society highlights transboundary, global risks. Complex systems emphasise interconnected infrastructures. Realism supports standardisation and technical harmonisation.	Risk is global and systemic, requiring international coordination and shared governance.

Integrating these perspectives into a structured risk governance framework addresses a gap in existing frameworks. Table 4 below presents a synthesis of several risk philosophies commonly held by stakeholders in the nuclear sector into a single table, following Table 3 above, specifically during decommissioning, along with their strengths and weaknesses as identified in the literature and how each of these perspectives impacts the overall risk governance process

**Table 4:** Strengths, Weaknesses and Impact of Philosophies of Risk

Philosophy	Strengths	Weakness	Impact on the Nuclear Sector	Example
1. Realist Perspective and Precautionary Risk	● Recognises inherent risks realistically. ● Balances caution with practicality. ● Encourages evidence-based mitigation.	● Can lead to over-caution, delaying necessary actions. ● May underplay social/ethical dimensions.	Prioritise public safety and environmental protection, leading to stricter regulations or nuclear phase-outs.	After the Fukushima disaster (2011), Germany phased out nuclear power through its Energiewende policy (Auer and Anatolitis, 2014). e.g. **Government and Regulatory Bodies**
2. Risk-Balancing	● Considers trade-offs between risks and benefits. Supports optimisation in decision-making. Aligns with pragmatic governance.	● May be perceived as compromising safety for cost/economics. ● Can lack clarity when values conflict.	Balance safety with economic and operational efficiency by investing in safer and more flexible nuclear technologies.	Development of Small Modular Reactors (SMRs) by companies like Rolls-Royce to improve safety and affordability (Rolls-Royce, 2023). e.g. **Industry and Operators**
3. Risk-Averse	● Prioritises safety and public trust. Reduces the likelihood of catastrophic events. Builds conservative designs.	● Leads to slow decision-making. ● Can stifle innovation and increase costs significantly.	Adopt a cautious approach due to high capital costs and policy uncertainty, requiring financial incentives to mitigate risks.	UK’s Hinkley Point C project required ‘Contracts for Difference’ to guarantee long-term returns (UK Department for Energy Security and Net Zero, 2013). e.g. **Investors and Financial Institutions**
4. Risk-Intolerant	● Ensures maximum precaution for safety-critical systems. ● Fosters public confidence in safety measures.	● Often unrealistic (zero residual risk is impossible). ● Results in high costs and operational inefficiency.	Public opposition to nuclear power influences policy decisions and can lead to project cancellations or phase-outs.	National referendums in Italy and Switzerland post-Chernobyl and Fukushima led to nuclear phase-outs (World Nuclear Association, 2011; World Nuclear Association, 2022). e.g. **Public and Civil Society**
5. Zero-Risk or Principle-Based	● Strong ethical grounding (e.g. ALARA principle). ● Appeals to moral arguments for absolute safety. ● Encourages strict compliance.	● Unachievable in practice for nuclear systems. ● Leads to paralysis by principle, delaying decommissioning or innovation.	Advocate against nuclear power due to accident risks and waste management concerns, influencing public opinion and policy.	Greenpeace’s campaigns have contributed to Belgium’s delayed plans to extend nuclear reactor lifespans (Greenpeace, 2023). e.g. **Environmental Groups**
6. Global Risk-Mitigation	● Promotes international collaboration and knowledge sharing. ● Addresses systemic, transboundary risks. ● Useful for the climate-nuclear nexus.	● Coordination challenges across jurisdictions. ● Can lead to diffused accountability and slow responses.	Promote international cooperation and best practices to enhance nuclear safety and reduce cross-border risks.	The International Atomic Energy Agency sets global safety standards and conducts peer reviews post-Fukushima (IAEA, 2023). e.g. **International Organizations**

The recognition that stakeholders operate within divergent philosophies of risk prompts a critical inquiry, which is relevant both to those in administrative positions of power and to researchers working on risk governance for nuclear decommissioning: Can risk governance evolve beyond a singular paradigm to embrace a genuinely pluralistic approach that integrates these diverse perspectives? If such an approach is conceivable, what inherent strengths might it possess? Could it foster deeper collaboration and more effective communication across the complex web of actors within the nuclear sector? Furthermore, might a systematic engagement with these differing risk philosophies enable the development of governance frameworks that are not only technically robust but also socially responsive and ethically grounded? Ultimately, does a nuanced understanding of these philosophical orientations offer a pathway toward a more holistic, adaptive and inclusive model of risk governance, one capable of addressing the inherently interdisciplinary challenges posed by nuclear decommissioning and long-term safety?

### New Perspective on Stakeholder Categorisation

**Fig. 1:**
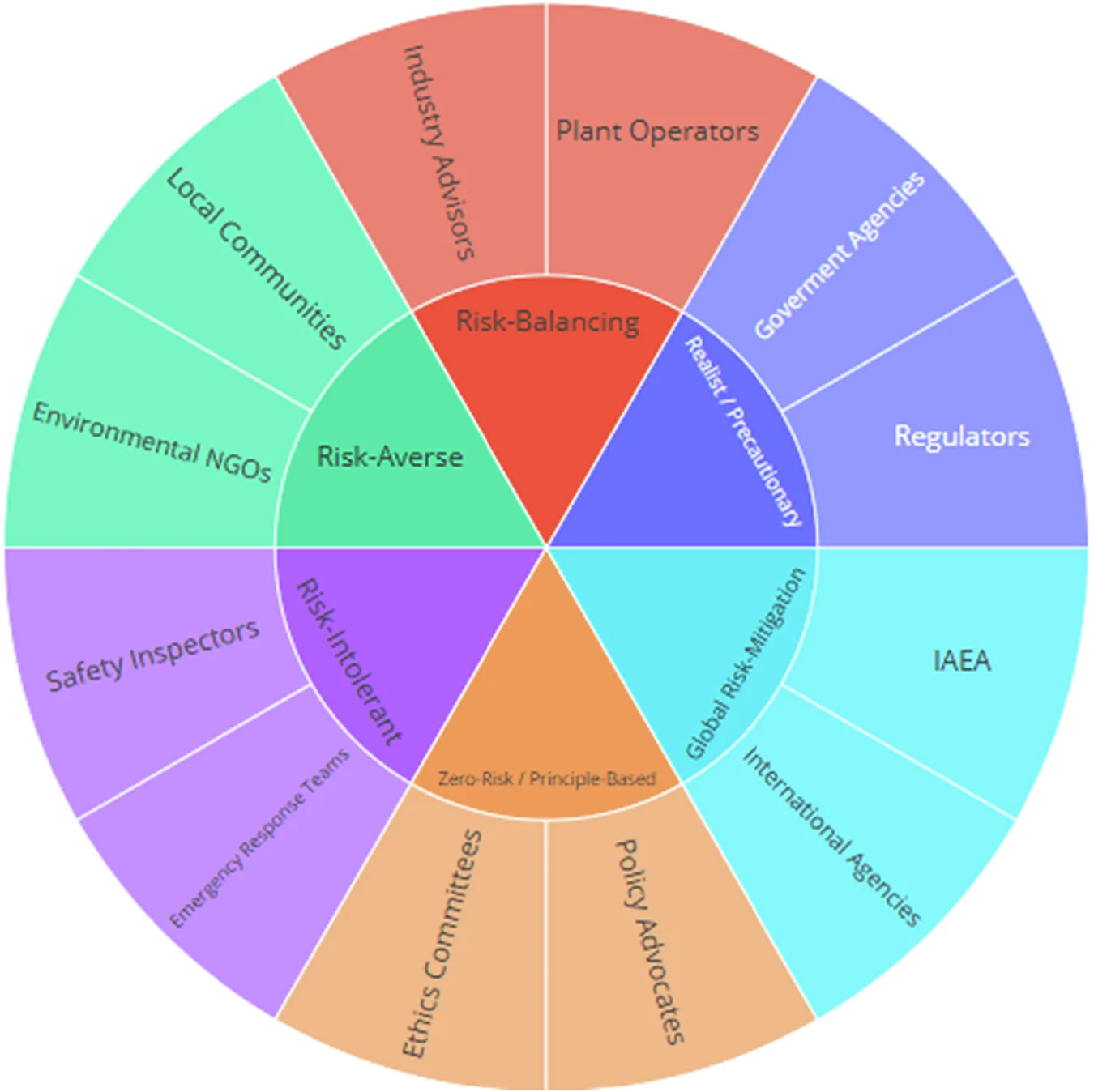
Risk Philosophy Categorisation of Nuclear Stakeholders

Existing stakeholder categorisations, discussed earlier in the paper, categorise stakeholders into roles (Table 1) and attitudes/functions (Table 2). There is less explicit attention, however, to defining stakeholders according to their risk philosophies. Yet, risk philosophy fundamentally influences how stakeholders perceive, assess and manage risk, as well as how they interact with one another (Renn, 2008; Slovic, 2010). Stakeholders who share similar risk philosophies, for example, are more likely to collaborate and build trust, whereas those with divergent philosophies may experience conflict or resistance in decision-making processes (Renn, 2008). Recognising these underlying perspectives is therefore essential for anticipating stakeholder behaviour and improving governance strategies in complex contexts such as nuclear decommissioning (Beck, 1992; Kasperson et al., 1988). Figure 1 below introduces a novel visualisation of stakeholder categorisation based on risk philosophy. This is not intended to replace existing categorisations, but rather to complement them by providing a deeper understanding of stakeholder philosophies and how this affects relational dynamics.

By combining the three categorisations as detailed in Tables 1, 2 and 4, a new classification system emerges which provides for a more holistic understanding. Table 4 presents a combined listing of three categorisation systems, with letters assigned for each category in Tables 1 and 2 and numbers assigned for each category in Table 4 (as indicated in each table). Using Tables 1, 2 and 4 and adopting a more pluralistic approach, we can classify the stakeholders depending on the situation or circumstance. As a simple example, we could define nuclear operators within the industry as PN2 *(Project developers, Normative stakeholders with a Risk Balancing philosophy)*. Table 5 provides further examples.

**Table 5:** Nuclear Stakeholder Classifications

Role (Table 1)	Function (Table 2)	Philosophy (Table 4)	Stakeholder Group
P	N	2	Industry & Operators
P	N	3	Investors, Banks, Private Equity
R	M	1	Regulatory Bodies, Local Governments
C	N	1	Ministries, Waste Management Organisations
C	N	2	Operators, Contractors
C	M	5	NGOs
I	N	1	Educators, Universities
I	M	1	Scientists, Technical Experts

## Discussion: The Pluralistic Approach to Risk Governance

Pluralism enables the triangulation of insights across these paradigms, promoting a more comprehensive and reflexive understanding of stakeholder concerns and governance challenges (Jasanoff, 1999; Klinke and Renn, 2012). This is particularly relevant in nuclear decommissioning, where technical uncertainties intersect with cultural values, ethical considerations and trust dynamics. By synthesising these perspectives, policymakers can design engagement strategies that are scientifically robust, socially legitimate and ethically defensible, thereby strengthening resilience and public trust in governance processes (OECD-NEA, 2019).

When considering various approaches to risk and risk philosophies, it is possible to identify the five core principles outlined below. These can be seen as a foundational principle-based approach to a normative ethics of a proposed model for risk governance in nuclear decommissioning, which the authors refer to as the *Pluralistic Approach to Risk Governance*.

A pluralistic approach to nuclear risk governance acknowledges that no single theoretical lens fully captures the complexity of risk perception, decision-making and governance in technologically advanced societies. Risk is inherently multidimensional, encompassing technical, social, cultural, ethical and psychological dimensions, requiring the integration of multiple frameworks to avoid reductionism (Renn, 2008; Stirling, 2007). For example, Beck’s Risk Society highlights systemic and transboundary risks, while Douglas and Wildavsky’s Cultural Theory explains value-based differences in public responses. Ethical frameworks address normative questions on how coherent and effective governance methods are, shaping how decisions are made and actions are taken, not only to improve justice but also to support broader goals such as accountability, legitimacy and responsible decision-making (Boudreau LeBlanc, 2023), while psychological theories illuminate cognitive biases shaping risk perception (Slovic, 2010).

### Application: A Principle-Based Approach for an Applied Normative Ethics

These principles constitute the normative and operational core of the framework, delineating the conditions under which it may achieve legitimacy and effectiveness. While the principles themselves are clearly articulated and underwrite the approach, the question of how they should be weighted, prioritised and balanced in practice remains an open and critical area for deliberation. It will depend on the viewpoints, values and disciplines involved.

The pluralism of risk enables it to be better understood, evaluated and managed in a variety of ways. The idea that risk is not a single, fixed, or solely technical term. Risk pluralism in nuclear decommissioning and governance is the understanding that stakeholders have varied definitions and perspectives on hazards, including social, cultural, psychological and ethical concerns, as well as technical considerations (See Operationalisation of the Pluralistic Approach below).

Still, there is less explicit attention to defining stakeholders based on their risk philosophy, which might differ across contexts depending on the strengths and weaknesses of the societal governance structure. The five principles below are of equal importance and their integration into a coherent pluralistic governance framework is critical to outlining the novelty of this approach compared to other formally proposed frameworks. The proposed pluralistic approach to risk governance will combine the five principles above to achieve a more adaptive and holistic approach. In addition, it will include the insights from the stakeholder philosophies proposed earlier in this paper.

### Principle 1: Rational Approach to Risk

This is the objective, evidence-based anchor of the framework. It ensures there is a baseline of risk assessment rooted in science, logic and quantitative analysis. Its purpose is to provide a common starting point from which diverse perspectives can interact without descending into relativism. It is used in nuclear decommissioning as in internationally accepted risk thresholds (e.g. IAEA standards), probabilistic safety assessments and radiological dose modelling as a baseline to evaluate hazards.

Using this principle, regulators, industry and experts anchor governance through evidence-based risk thresholds and scientific modelling. This provides a shared technical baseline that legitimises decisions and ensures compliance, even if contested by other perspectives. However, it’s important to recognise that this is a necessary principle but not sufficient in itself.

### Principle 2: Stakeholder Proportionality

This is recognising that risk is not experienced equally. Different stakeholders (e.g. local communities, regulators, unions, scientists) interpret and emotionally respond to risks based on history, culture, values and lived experience. Its purpose is to legitimise and include diverse risk perceptions as valid inputs, even if they challenge technical data. In nuclear decommissioning, it is important to acknowledge the fears of local communities living near legacy waste, even if technical models say the risk is low and design participatory forums to capture those perceptions.

Perception in itself is not misinformation. Rather, it reflects lived experience. Although perception may not be based on objectivity, it is a key influence on how smoothly nuclear decommissioning takes place in a specific context. Using this principle, local communities, NGOs and civil society actors bring lived experience and cultural meaning to risk, which must be treated as valid and evaluated alongside technical data. Recognising perceptions builds social licence, reduces contestation and prevents delays caused by mistrust.

### Principle 3: Fairness in Risk Distribution

Here, we recognise that no risk governance process can eliminate all risk. The challenge is to decide what is acceptable and to whom. Then ensure that discussions about tolerable risk are not only technical but also ethical and political. In nuclear decommissioning, this principle can be used by governments, regulators and industry to address who bears the residual risks of decommissioning, including spatial and intergenerational equity. This principle ensures ethical accountability and prevents unjust burden-shifting onto vulnerable or future populations, where acceptability is a value judgment, not just a scientific conclusion.

This requires making the distribution of residual risks explicit, including who is exposed, who benefits and who has decision authority, rather than treating ‘acceptability’ as a purely technical threshold. It also means embedding ethical and political deliberation into formal decision processes, so that judgments about tolerability reflect societal values as well as expert assessments. In nuclear decommissioning, this strengthens accountability by ensuring that neither local communities nor future generations are implicitly assigned disproportionate risk burdens without transparent justification.

### Principle 4: Optimising Risk to Achieve ALARP

It is important to recognise that all action carries risk, including inaction. Governance must weigh risks of intervention (e.g. moving radioactive waste) against risks of passivity (e.g. long-term containment failure) to ensure the risk is as low as reasonably practicable (ALARP). This principle allows room for nuanced decision-making that recognises competing goods: innovation vs safety, economic growth vs precaution, urgency vs deliberation. Using this principle, operators, investors, governments and communities all face competing priorities between cost, safety, innovation and local benefits.

By openly acknowledging trade-offs, governance prevents stalemates and fosters transparent, negotiated compromises. In nuclear decommissioning, operators, investors, governments and communities might ask themselves when they should dismantle the nuclear power plant versus contain nuclear waste on site. When do they invest in new technologies vs maintain proven but outdated ones? It is important to note that governance is not about *avoiding* risk but about *managing* competing risks transparently.

### Principle 5: Recognition of Randomness and Uncertainty

Complex systems (technical, ecological, social) behave in nonlinear and unpredictable ways. Risk governance must embrace the limits of prediction. To prevent overconfidence in models, we must encourage adaptive learning and build resilience. In nuclear decommissioning, scientists, regulators, communities and international organisations must embrace unpredictability, integrate scenario planning and sensitivity analysis and ensure suitable safety margins (which provide assurance against Beyond Design Basis Events). The principle encourages monitoring long-term systems dynamically rather than relying on static compliance. Recognising accuracy and uncertainty helps avoid false assurances and builds long-term trust in decommissioning governance.

### Operationalisation of the Pluralistic Approach

In practical terms, operationalising pluralism means understanding why different stakeholders interpret, prioritise and respond to risk differently in the first place, rather than simply identifying stakeholders and providing opportunities for participation. Differences in stakeholder responses are rooted in their underlying philosophies, namely, their epistemological assumptions (what counts as valid knowledge), axiological commitments (what is valued or considered acceptable) and teleological orientations (what outcomes are seen as desirable).

Without explicitly incorporating these philosophies, pluralism risks remaining superficial, with stakeholder engagement reduced to a checklist exercise rather than a genuine integration of perspectives. In contrast, recognising stakeholder philosophies enables governance systems to map, compare and reconcile fundamentally different risk logics, such as precautionary, cost-benefit, rights-based or systems-oriented approaches. It achieves this by translating abstract stakeholder philosophies into structured governance components. Specifically, it systematically categorises stakeholders based on their underlying epistemological, axiological and teleological orientations and then aligns these with a defined set of guiding principles (e.g. fairness, proportionality, ALARP and uncertainty recognition). This creates a clear linkage between who the stakeholders are, how they understand risk and how decisions should be made. By doing so, the approach provides a mechanism for comparing, reconciling and integrating competing perspectives within a single decision-making framework, enabling transparent trade-offs, consistent reasoning and practical application in governance processes rather than remaining at a purely conceptual level.

This pluralistic framework is applicable across institutions such as the International Atomic Energy Agency and the International Risk Governance Council, as well as national regulators, decommissioning authorities and environmental planning bodies and can enhance key decision-making processes, including nuclear decommissioning strategy selection, radioactive waste management and disposal design, site selection, stakeholder engagement and long-term energy and investment planning. In the UK context, the framework applies to institutions such as the UK’s nuclear regulators and governance bodies, including the Office for Nuclear Regulation, the Environment Agency and the Nuclear Decommissioning Authority, as well as organisations such as Sellafield Ltd.

## Conclusions

Different nuclear stakeholder groups often have varying philosophies of risk due to their unique interests, responsibilities, values and degrees of vulnerability to the consequences of risk. These differences in philosophy often reflect the diverse ways in which each group assesses, manages and responds to risk. These philosophies reflect not only technical assessments of danger or probability but also what matters most to different actors: their priorities, power dynamics, cultural context and moral frameworks. When well understood, these theoretical frameworks of risk can help practitioners systematically explore and explain risks from these multiple viewpoints, enhancing the rigour and effectiveness of risk assessment and management practices during decommissioning.

Adopting the identified stakeholder philosophies complementarily rather than in isolation is justified because no single perspective is sufficient to capture the full complexity of nuclear risk governance. Each philosophy is grounded in distinct epistemological and normative assumptions; some prioritise technical rationality, others emphasise social construction, ethical justice, uncertainty, or systemic complexity. When applied individually, these perspectives tend to yield partial, potentially biased interpretations of risk, overlooking critical dimensions such as stakeholder values, distributional impacts and deep uncertainty.

A complementary approach mitigates these limitations by allowing the strengths of one perspective to offset the weaknesses of another. Moreover, stakeholders themselves implicitly operate from different philosophical positions. Treating these philosophies as complementary reflects this real-world plurality, enabling governance frameworks to better align with diverse expectations and values. This, integrated within the five principles, in turn, enhances legitimacy, inclusivity and decision quality by avoiding the privileging of a single worldview and facilitating more transparent, adaptive decision-making.

Each risk philosophy offers valuable insights and methods for approaching the proposed pluralistic nuclear risk governance. By combining elements from these philosophies, policymakers can create a more comprehensive and inclusive framework for managing nuclear risks. The idea here is not to debate the merits of competing paradigms and metatheoretical orientations, for these only entrench each point of view within its principles, or to make one theory superior over another, which can lead to biased, rigid and oversimplified analyses that fail to adapt to the complexities of real-world issues, thereby limiting the effectiveness of risk governance in waste disposal facilities and other decision-making processes. Rather, the aim was to bring a comprehensive approach to the risk management problem with these diverse perspectives, which leads to a more comprehensive and holistic approach to stakeholder management, risk governance and nuclear waste management.

The proposed Pluralistic Approach is a multi-faceted approach that incorporates these diverse philosophies and five integrated principles that can help policymakers address nuclear risks’ technical, social, ethical and economic dimensions, leading to more resilient and responsible waste management and risk governance strategies via five principles identified and the nuclear stakeholder classification system. These principles constitute the normative and operational core of the framework, delineating the conditions under which it may achieve legitimacy and effectiveness. While the principles themselves are clearly articulated and underpin the approach, the question of how they should be weighted, prioritised and balanced in practice remains an open and critical area for deliberation during operationalisation, as this is likely to vary across contexts such as national regulatory regimes, socio-political and cultural settings, technological configurations and the specific stakeholder landscape involved.

The principles developed in this study are primarily situated within the context of nuclear decommissioning, where issues of long-term uncertainty, intergenerational responsibility and stakeholder sensitivity are particularly pronounced. However, the underlying stakeholder philosophies and pluralistic principles are not confined to this domain. Rather, they reflect broader patterns of risk interpretation, value formation and decision-making that are inherent across the wider nuclear risk governance landscape, including reactor operation, new build and lifecycle management. This wider applicability is justified by the fact that nuclear governance across all phases involves the same fundamental tensions between technical uncertainty, social perception, ethical responsibility and institutional constraints. Consequently, the pluralistic framework provides a transferable analytical lens that can support more integrated, adaptive and legitimate governance across different stages of the nuclear lifecycle, rather than being limited to decommissioning-specific contexts.

The novelty of this study lies in its development of an integrated five-principle pluralistic framework that explicitly incorporates and operationalises multiple philosophical dimensions, epistemological, axiological and teleological perspectives, within a single coherent approach to risk governance, moving beyond existing models of procedural inclusion to enable substantive integration of diverse knowledge systems, values and motivations in decision-making.

## Data Availability

No datasets were generated or analysed during the current study.
